# FLJ10540 is associated with tumor progression in nasopharyngeal carcinomas and contributes to nasopharyngeal cell proliferation, and metastasis via osteopontin/CD44 pathway

**DOI:** 10.1186/1479-5876-10-93

**Published:** 2012-05-16

**Authors:** Chang-Han Chen, Li-Yen Shiu, Li-Jen Su, Chi-Ying F Huang, Shun-Chen Huang, Chao-Cheng Huang, Yu-Fang Yin, Wei-Sheng Wang, Hsin-Ting Tsai, Fu-Min Fang, Wan-Chu Chuang, Hong-Chang Kang, Chung-Feng Hwang

**Affiliations:** 1 Center for Translational Research in Biomedical Sciences, Kaohsiung Chang Gung Memorial Hospital, Kaohsiung, Taiwan; 2 Department of Otolaryngology, Kaohsiung Chang Gung Memorial Hospital, Chang Gung University College of Medicine, Kaohsiung, Taiwan; 3 Kaohsiung Chang Gung Head and Neck Oncology Group, Kaohsiung Chang Gung Memorial Hospital, Kaohsiung, Taiwan; 4 The Department of Medical Research and Development, Show Chwan Memorial Hospital and Chang Bing Show Chwan Memorial Hospital, Changhua, Taiwan; 5 Graduate Institute of Systems Biology and Bioinformatics, National Central University, Jhongli, Taiwan; 6 Institute of Clinical Medicine, National Yang-Ming University, Taipei, Taiwan; 7 Department of Pathology, Kaohsiung Chang Gung Memorial Hospital, Chang Gung University College of Medicine, Kaohsiung, Taiwan; 8 Department of Radiation Oncology, Kaohsiung Chang Gung Memorial Hospital, Chang Gung University College of Medicine, Kaohsiung, Taiwan; 9 Department of Otolaryngology, Kaohsiung Chang Gung Memorial Hospital, Chang Gung University College of Medicine, No. 123, Ta-Pei Road, Niao Sung District, Kaohsiung 833, Taiwan

**Keywords:** Nasopharyngeal carcinoma, FLJ10540, Osteopontin, CD44

## Abstract

**Background:**

Nasopharyngeal carcinoma (NPC) is well-known for its highly metastatic characteristics, but little is known of its molecular mechanisms. New biomarkers that predict clinical outcome, in particular the ability of the primary tumor to develop metastatic tumors are urgently needed. The aim of this study is to investigate the role of FLJ10540 in human NPC development.

**Methods:**

A bioinformatics approach was used to explore the potentially important regulatory genes involved in the growth/metastasis control of NPC. FLJ10540 was chosen for this study. Two co-expression strategies from NPC microarray were employed to identify the relationship between FLJ10540 and osteopontin. Quantitative-RT-PCR, immunoblotting, and immunohistochemistry analysis were used to investigate the mRNA and protein expression profiles of FLJ10540 and osteopontin in the normal and NPC tissues to confirm microarray results. TW01 and Hone1 NPC cells with overexpression FLJ10540 or siRNA to repress endogenous FLJ10540 were generated by stable transfection to further elucidate the molecular mechanisms of FLJ10540-elicited cell growth and metastasis under osteopontin stimulation.

**Results:**

We found that osteopontin expression exhibited a positive correlation with FLJ10540 in NPC microarray. We also demonstrated comprehensively that FLJ10540 and osteopontin were not only overexpressed in NPC specimens, but also significantly correlated with advanced tumor and lymph node-metastasis stages, and had a poor 5-year survival rate, respectively. Stimulation of NPC parental cells with osteopontin results in an increase in FLJ10540 mRNA and protein expressions. Functionally, FLJ10540 transfectant alone, or stimulated with osteopontin, exhibited fast growth and increased metastasis as compared to vehicle control with or without osteopontin stimulation. Conversely, knockdown of FLJ10540 by siRNA results in the suppression of NPC cell growth and motility. Treatment with anti-CD44 antibodies in NPC parental cells not only resulted in a decrease of FLJ10540 protein, but also affected the abilities of FLJ10540-elicited cell growth and motility in osteopontin stimulated-NPC cells.

**Conclusions:**

These findings suggest that FLJ10540 may be critical regulator of disease progression in NPC, and the underlying mechanism may involve in the osteopontin/CD44 pathway.

## Background

Nasopharyngeal carcinoma (NPC), the most common head and neck cancer, is a disease in which malignant cells form in the epithelium of the nasopharyngeal cavity. There is a high incidence of NPC in southern China and Southeast Asia, where the annual incidence is more than 20–30 cases per 100,000 people [1], and this pattern persists in those who have emigrated. The etiology of NPC seems to follow a multistep process, in which EBV infection, ethnic background, consumption of food and alcohol, and environmental carcinogens all seem to play a role [2]. Advances in diagnostic imaging, radiation therapy, and concurrent chemoradiotherapy have achieved better locoregional control, however final treatment outcomes are not satisfactory [3,4].

The process of metastasis which is considered a late event in tumorigenesis is very complex. Due to the rapid growth behavior of NPC, it has a high tendency to invade adjacent regions and metastasize to regional lymph nodes and distant organs [5-7]. More than 60% of NPC patients are first diagnosed with metastasis, leading to a high rate of treatment failure [6]. The prognosis of NPC depends primarily on clinical TNM staging and tumor size [8]. However, NPC patients with the same clinical stage often have different clinical courses, suggesting that TNM staging and tumor size are insufficient to accurately predict prognosis [9]. Thus it is important to search for new therapeutic targets and a better understanding of the mechanisms involved in the spread of NPC.

Osteopontin, also known as SPP-1, was first detected in 1979 from malignantly transformed epithelial cell lines and T cells [10]. Human osteopontin is composed of 314 amino acid residues containing two crucial functional domains, the calcium-binding domain and arginine-glycine-aspartic acid (RGD) sequences [11]. It is a multifunctional cytokine which mediates in bone resorption, atherosclerosis, angiogenesis, wound repair, immune function, tissue injuries, and disease formation [12,13]. Osteopontin expression has been associated with cancer markers for many years [14]. Strong osteopontin expression in tumor cells has been shown in a variety of cancer types and is associated with poor prognosis and metastasis including gastric, ovarian, lung, breast, prostate, liver, sarcomas, colon, renal, and head and neck cancers [15-18]. However, the precise role of osteopontin in tumor development and promoting cancer metastasis in NPC is largely unknown.

FLJ10540 is also known by other names, including CEP55 [19], and C10orf3 [20]. FLJ10540 is overexpressed in human colon cancer [20], hepatocellular carcinoma (HCC) [21], lung cancer [22] and oral cavity squamous cell carcinoma (OCSCC) [23], suggesting that it may function as an oncogenic characteristics in tumor development. However, there is little information regarding the expression and significance of FLJ10540 with tumor histological grade, stage, and patient outcome in NPC patients. In addition, the precise roles of FLJ10540 in tumor growth and metastasis in NPC are large unknown. The purpose of the present study was to examine the expression levels of FLJ10540 in NPC specimens, and to correlate the results with clinicopathologic variable survival and to investigate the functional role of FLJ10540 in human NPC development.

## Methods

### Patients and tumor samples

To measure the expression of the FLJ10540 and osteopontin transcripts in NPC tissues, fresh tissues were obtained from patients with NPC biopsy specimens. Controls included fresh normal nasopharyngeal mucosal tissues from other patients with biopsies for other non-neoplastic diseases. These materials were histologically confirmed by frozen sections before quantitative RT-PCR and western blotting assays. Available paraffin-embedded tissue blocks were retrieved from 63 NPC patients. Clinicopathological information for each subject, including gender, age, tumor-(T) stage, nodal-(N) status, TNM stage, and disease-specific survival (DSS), was obtained retrospectively from clinical records and pathology reports. NPC patients received local head and neck examinations before treatment, along with staging examinations, including whole body bone scans, abdominal ultrasonography, computed tomography, and/or magnetic resonance imaging. Using the 2002 American Joint Committee on Cancer staging system, 19 patients were classified as T1, 25 as T2, four as T3, and 15 as T4. Twenty-eight patients were classified as N0, 13 as N1, 18 as N2, and four as N3. Twelve patients were determined to be at stage I, 20 at stage II, 11 at stage III, and 20 at stage IV. The method of radiotherapy for NPC was, in general, uniform throughout this period. All patients were regularly monitored after receiving radiotherapy and/or chemotherapy until death or their last appointment, according to the intervals and protocols of follow-up as detailed in a previous study [3]. Survival data were either obtained from the cancer registry of our hospital or collected from the patients’ attending physicians. Locoregional failure was determined by pathological diagnosis or progressive deterioration, as shown on consecutive imaging studies. To identify distant metastases, patients underwent chest radiography yearly and bone scans or abdominal ultrasonography whenever indicated. All study participants provided informed consent, and study design was approved by the Medical Ethics and Human Clinical Trial Committee of Chang Gung Memorial Hospital.

### RNA extraction, and quantitative RT-PCR (Q-RT-PCR)

Tissue samples were frozen in liquid nitrogen and stored at −80°C prior to RNA extraction. The RNA extraction, and quantitative RT-PCR assays were performed as described previously [4]. For Q-RT-PCR, *FLJ10540* and *osteopontin* Taq-Man probes (ABI) were used. Data were represented as mean ± s.d. To analyze the distribution of normal and tumor areas, we used the Wilcoxon signed rank test between two groups for statistical analysis. A *P*-value of less than 0.05 was considered statistically significant. *GAPDH* was used as an internal control for comparison and normalization of the data. Assays were performed in triplicate using an Applied Biosystems Model 7500-Fast instrument.

### Immunoblot analysis

For tissue protein extraction, frozen samples were homogenized in RIPA lysis buffer (50 mm Tris–HCl, pH 7.5, 150 mm NaCl, 1% NP-40, 0.5% Na-deoxycholate, and 0.1% SDS). The western blotting assay was performed as described previously [4]. The antibodies used in this study included polyclonal antibodies against FLJ10540 (generated by us) [23], HA (3F10, Roche Biochemicals, Indianapolis, IN, USA), and β-actin (monoclonal; Santa Cruz Biotechnology, Santa Cruz, CA, USA). The proteins were investigated using X-ray films.

### Immunohistochemical study

Normal and tumor NPC tissue samples were selected by a pathologist based on diagnosis and microscopic morphology. Immunohistochemical staining was performed as described previously [4,23,24]. After antigen retrieval, the sections were incubated with diluted anti-FLJ10540 antibody (polyclonal; generated by us; 1:200; polyclonal; Abnova, Taiwan 1:100), and anti-osteopontin (polyclonal; Abnova, Taiwan 1:100; polyclonal; Santa Cruz Biotechnology, Santa Cruz, CA, USA; 1:50 at room temperature for 1 hour, followed by washing with PBS. Horseradish peroxidase/Fab polymer conjugate (PicTure™-Plus kit; Zymed, South San Francisco, CA, USA) was then applied to the sections for 30 min followed by washing with PBS. Finally, the sections were incubated with diaminobenzidine for 5 min to develop the signals. A negative control was run simultaneously by omitting the primary antibody. The reactivity level of the immunostained tissues was evaluated independently by two pathologists who were blind to the subjects’ clinical information. Between 15 and 20 high-power fields were viewed. Criteria were developed for quantitating the immunoreactivities of the osteopontin staining in both the normal and tumor sections using a score range of 0 to +3, where 0 indicated no positive cell staining, +1 less than 5% positive cell staining, +2 5-50% positive cell staining, and +3 more than 50% positive cell staining. Similarly, the stain intensity was graded as +0, +1, +2, or +3 as previously described [25]. The quantitating of the immunoreactivities of the FLJ10540 staining followed the protocol of osteopontin. High-expressions of FLJ10540 and osteopontin were defined as +2 or higher for both scoring methods.

### Cell culture, establishment of stable clones, gene silencing using siRNA, promoter plasmids, and luciferase assays

NPC-TW01 and Hone-1 cell lines derived from primary nasopharyngeal tumors of untreated NPC patients were used for functional assays [26-28]. All cell culture-related reagents were purchased from Gibco-BRL (Grand Island, NY, USA). TW01 cells were grown in DMEM, however the Hone1 cells were grown in RPMI containing 10% FBS and 100 U/ml penicillin and streptomycin (Gibco-BRL). HA-vector (pcDNA3.1), and HA-FLJ10540 were transiently transfected into cancer cells using Lipofectamine (Invitrogen) according to the manufacturer’s instructions. TW01 and Hone1 cells stably expressing FLJ10540 were selected with 400 μg/ml G418 (Calbiochem Novabiochem, San Diego, CA, USA). The cells were then harvested and analyzed for exogenous FLJ10540 expression by Western blotting. 5’-upstream fragments of *FLJ10540* gene was amplified from human genomic DNA and verified by sequencing. The PCR fragments were cloned into firefly luciferase reporter vector pGL3-Basic (Promega) NheI and HindIII sites which were designed into the forward and the reverse primers, respectively. pRL-TK (Promega), a *Renilla* luciferase internal control vector, was used for normalization of firefly luciferase signals. For co-transfection experiments, TW01 cells were co-transfected with 100 ng firefly luciferase reporter plasmids (pGL3-Basic or pGL3-FLJ10540) and 10 ng of pRL-TK *Renilla* luciferase internal control plasmid. After 24h, the cells were incubated with serum-free medium for 18h after osteopontin was added. The Luciferase activity was measured using Dual Glo™ Luciferase Assay System (Promega), One double-stranded synthetic RNA oligomers (5’-GGGAGAAATTGCACACTTAtt-3’; Ambion) deduced from human *FLJ10540* and one negative control siRNA (#4611G; Ambion) were used in the siRNA experiments. The specific protein levels were determined by Western blotting analysis with specific antibodies.

### Cell viability assay

The viability of sub-confluent cells was analyzed by 3-(4,5-dimethylthiazole-2-yl)-2,5-diphenyltetrazolium bromide (MTT) reduction assay. The assay was performed in 96-well plates seeded with 4000–6000 cells/well in 200 μl. After 24 hours, 100 μl of culture medium containing 25 μl MTT solutions was added (1 g MTT (Sigma M5655) dissolved in 200 mL D-PBS). It was stored for 4 hours at 37°C, and then 100 μl DMSO buffer was added and left protected from light for 10 min. Readout was performed using a microplate reader (Labsystems Multiscan MCC/340) at 540 nm. Absorbance at 692 nm was used as the reference.

### Migration and invasion assays

Migration and invasion assays were conducted with TW01-/Hone1-vehicle, TW01-/Hone1-FLJ10540, TW01-negative, and TW01-siFLJ10540 stable clones using 24-well Transwell chambers (8-μm pore size polycarbonate membrane; Costar). The migration and invasion assays were performed as described previously [4,23,24]. Briefly, cell migration and invasion were evaluated by counting the number of TW01-/Hone1-vehicle, TW01-/Hone1-FLJ10540, TW01-negative, and TW01-siFLJ10540 stable clone cells that had migrated or invaded by 200X phase-contrast microscopy on three independent membranes, then normalized against the vehicle cells to determine the relative ratio.

### Microarray analysis, data analysis and clustering algorithm

The microarray data of NPC (GEO Series accession number is GSE12452) has been deposited in NCBIs Gene Expression Omnibus (GEO) and were analyzed using GeneSpring® 7.3.1 software (Silicon Genetics, Redwood City, CA). The microarray data was normalized by Robust Multichip Average (RMA) algorithm to produce and normalize the Affymetrix expression signals for the transcripts based on corresponding probe pairs of oligonucleotides. One-way analysis of variance (ANOVA) was used to build an explicit model about the sources of variances that affect the measurements. The values of *p*< 0.05 were considered as statistically significant.

### Statistical analyses

Several clinicopathological factors were evaluated, including gender, age, T stage, N status, and TNM stage. Fisher’s exact test was used to evaluate the correlation between the clinicopathological variables and the expressions of FLJ10540 and osteopontin. A p value of less than 0.05 was considered to indicate statistical significance in all analyses. Clinicopathological variables and the expression of FLJ10540 and osteopontin were taken into account for the analysis of survival, based on the Kaplan-Meier method; statistical significance, defined as a p value of less than 0.05, was assessed by log-rank test. To determine the effect of particular prognostic factors on survival, a multivariate analysis was performed according to Cox’s regression model.

## Results

### Elevated FLJ10540 expression in NPC tissues

**Figure 1:**
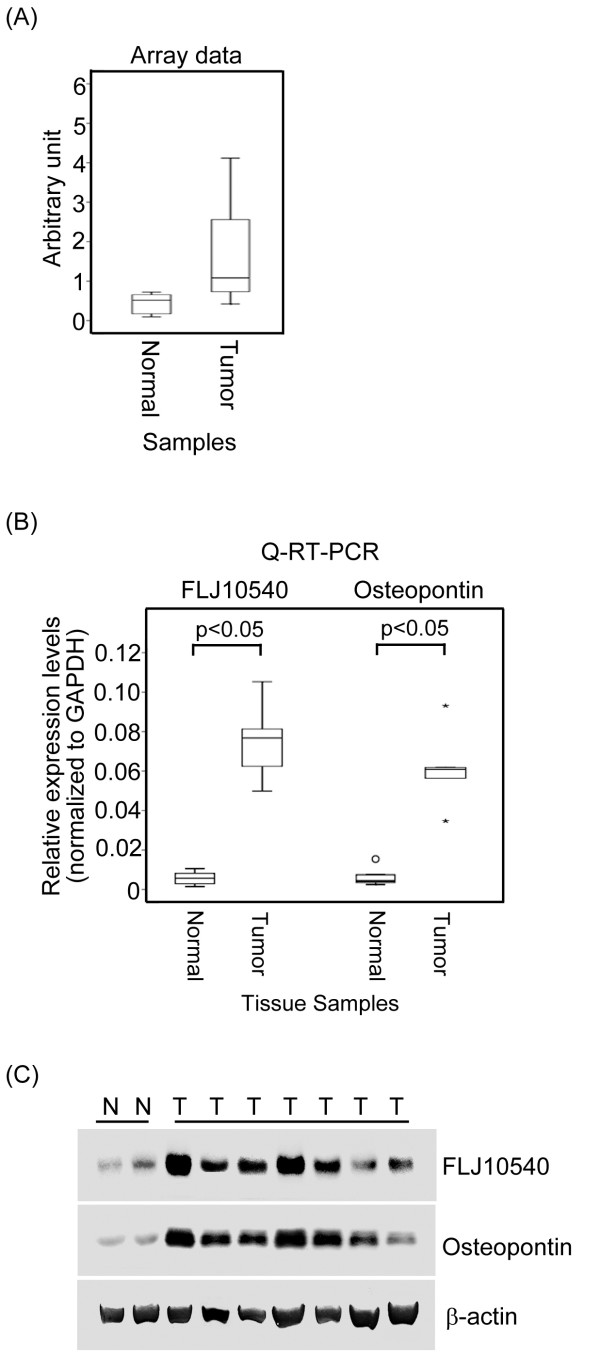
**FLJ10540 was highly correlated with osteopontin expression in human NPC specimens.** (**A**) The microarray expression patterns of *FLJ10540* in NPC patients were normalized against the expression patterns on HG_U133 plus 2.0 chips. The boxplot shows the data distribution as a grouping classification and indicates that there was a statistically significant difference (*p*<0.0001) between the tumor tissues and the normal tissues from NPC patients. (**B**) The mRNA expression levels of *FLJ10540* and *osteopontin* from twelve NPC patients and five normal tissues were determined by Q-RT-PCR. The results were normalized against the expression levels of *GAPDH* mRNA in each sample. N: normal tissues; T: tumor tissues. (**C**) Western blotting analysis of FLJ10540 and osteopontin expressions were performed in seven NPC samples and two normal tissues. Total proteins were extracted from respective tissues and probed with antibodies against FLJ10540 and osteopontin. β-actin was used as a control.

A significant challenge in the post-genomic era is determining how to prioritize differentially expressed and poorly characterized targets in cancer-related microarray profiling studies. This study aimed to identify and characterize novel oncogenes that are only conserved in vertebrates, but not in invertebrates, based on the hypothesis that these novel genes may possess unique function in human cancer development. We used a bioinformatics approach to search for potentially important regulatory genes involved in growth control of human NPC. The publicly accessible microarray data has been deposited in NCBIs Gene Expression Omnibus (GEO) but can be accessed through the GEO Series accession number GSE12452 [29]. According to the microarray data of 31 primary NPC specimens, 592 out of 54,675 differentially expressed transcripts between normal and tumor areas were clustered by the ANOVA (*p*<0.05) based on three-fold differences and tested using the Benjamini and Hochberg false discovery rate correlation (*p*<0.05). These molecular portraits, containing 193 up-regulated and 399 down-regulated transcripts, could successfully distinguish the patients’ normal and tumor areas by hierarchical clustering ( Additional file 1: Figure S1). As a result, we focused on the poorly characterized gene, *FLJ10540*. *FLJ10540* exhibited up-regulated expression patterns in NPC specimens in microarray database (Figure 1A). To validate the gene expression profile data for *FLJ10540*, Q-RT–PCR was performed on 12 NPC-tumor and five normal tissue samples. Figure 1B shows that overexpression of *FLJ10540* was observable in our NPC patient samples. This result raises the possibility that upregulation of *FLJ10540* may lead to the abnormalities found in human NPC.

### Using the concept of syn-expression to uncover osteopontin, as the upstream regulator of FLJ10540 in NPC specimens

Using the concept of syn-expression, genes belonging to the same pathway often show the same regulatory profiles under a variety of biological situations [30]. Hence, we employed the same publicly accessible microarray dataset, as described earlier, to gain insight into the functional concordance of co-expressed genes. To test this hypothesis, we first examined whether the mRNA expression profile of *FLJ10540* correlated with any ligand in the publicly accessible microarray database of NPC. Of these co-expressed genes, the top candidate was *osteopontin,* which was highly positively correlated with the mRNA expression level of *FLJ10540* in NPC cancer patients (r=0.72 *p*<0.001). This raises the possibility that these two molecules are functionally linked or in the same pathway.

The same panels of 12 NPC-tumor and five normal tissues were also applied to examine the mRNA expression level of *osteopontin* by Q-RT-PCR. As shown in Figure 1B, the mRNA expression levels of *osteopontin* were increased in the tumor samples as compared to those in normal tissue samples. The expressions of FLJ10540 and osteopontin in another batch of freshly frozen tumor and normal samples were further verified by western blotting. Our data indicated that the western blot results were consistent with the observed mRNA expressions of FLJ10540 and osteopontin in NPC specimens (Figure 1C). Taken together, these results suggest that there is a significant positively correlation between FLJ10540 and osteopontin in human NPC.

**Figure 2:**
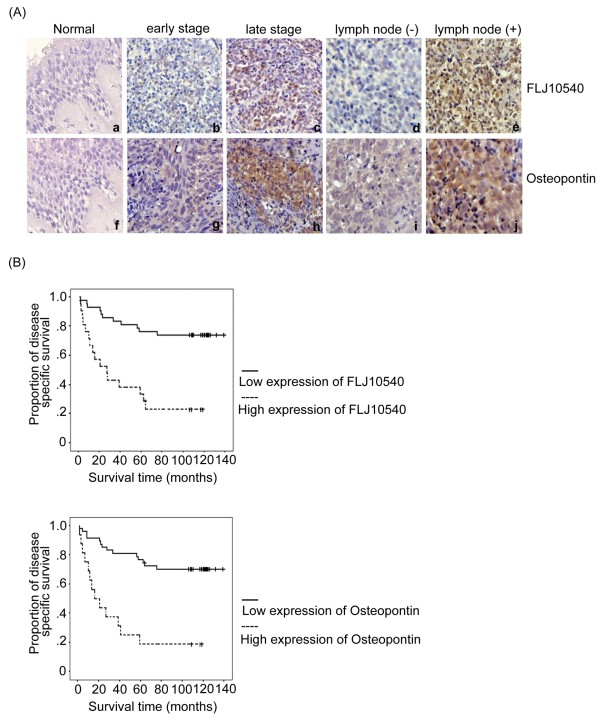
**Immunohistochemical staining for FLJ10540 and osteopontin and overall survival in patients with NPC.** (**A**) The FLJ10540 and osteopontin intensities in various tissues were evaluated by immunohistochemical staining. FLJ10540 and osteopontin immunohistochemical staining intensities are shown in tumor and normal tissues. Normal tissues displayed very weak FLJ10540 and osteopontin staining in the cytoplasm (a, and f). The stainings for FLJ10540 and osteopontin were higher in tumor tissues (b-e and g-j). Significant cytoplasm expressions of FLJ10540 and osteopontin were observed in early (b, and g) and late (c, and h) stage tumors. Original magnification ×100. (**B**) The survival period of those patients (n=63) with strong expressions of FLJ10540 and osteopontin (dashed lines) were significantly shorter than for those with lower expressions of FLJ10540 and osteopontin (solid line). The difference in survival was statistically significant (*p*<0.001) according to the log-rank test.

To investigate the clinical implications of FLJ10540 and osteopontin in NPC, immunohistochemical analysis was performed to determine the expressions and distributions of FLJ10540 and osteopontin in 63 human NPC samples and 10 normal tissues. The representative results of FLJ10540 and osteopontin immunostaining in NPC are shown in Figure 2A. First, FLJ10540 and osteopontin revealed very weak but detectable staining in the epithelium (Figure 2A, a and f). In the tumor samples, FLJ10540 and osteopontin expressions were positively correlated with tumor stage and node stage of the tumor cells (Figure 2A, b-e and g-j). However, strong stainings were not observed in tumor tissues when anti-FLJ10540 and anti-osteopontin antibodies were pre-incubated with protein corresponding to a portion of FLJ10540 and osteopontin, respectively (data not shown). Third, FLJ10540 and osteopontin were largely localized in the cytoplasm of the tumor sample cells as well as in normal tissues. To further confirm our immunohistochemisty results, anti-FLJ10540 and anti-osteopontin antibodies from Abnova produced almost the same results. In addition, χ^2^ test was done to assess the association between FLJ10540 and osteopontin. As shown in Table 1, FLJ10540 again was significantly associated with osteopontin. Taken together, these results suggest that patients with a high FLJ10540 score had a significantly higher osteopontin score in human NPC.

**Table 1:** χ²test was done to assess the association between FLJ10540 and osteopontin

	**Osteopontin expression**		***p-*value**^**a**^
	**Low**	**High**	
FLJ10540 expression			<0.001
Low	40	2	
High	7	14	

^a^*p*-value from Chi-square test. Statistically significant (*p* < 0.05).

### Prognostic value of FLJ10540 and osteopontin expression levels in NPC

As shown in Table 2, patients with stage III-IV tumors, a higher rate of lymph node metastasis, and TNM stages III-IV had significantly higher expressions of FLJ10540 or osteopontin compared with patients with stage I-II tumors (*p<*0*.*001), with no or a lower rate of lymph node metastasis (*p*=0*.*001 and *p=*0*.*007), and TNM stages I/II (*p<*0*.*001). No significant differences between high and low levels of FLJ10540 and osteopontin were observed in terms of the patients’ age, gender, or histological type at the time of diagnosis. These findings suggest that both FLJ10540 and osteopontin expressions correlate with higher tumor progression from an early stage to advanced stage.

**Table 2:** Comparisons of FLJ10540 and osteopontin expressions between the subgroups of various clincopathological parameters

	**FLJ10540 expression**		***p-*value**^**a**^	**Osteopontin expression**		***p-*value**^**a**^
	**Low**	**High**		**Low**	**High**	
	**(n=42)**	**(n=21)**		**(n=47)**	**(n=16)**	
Age (year),			0.582			0.212
< 50	17	7		20	4	
≥ 50	25	14		27	12	
Gender			0.838			1.000
Male	31	16		35	12	
Female	11	5		12	4	
Histology			0.856			0.816
WHO type II	25	12		28	9	
WHO type III	17	9		19	7	
Tumor size			<0.001			<0.001
T1-T2	40	4		40	0	
T3-T4	2	17		3	16	
Nodal stage			0.001			0.007
N0-N1	34	7		35	6	
N2-N3	8	14		12	10	
AJCC staging			<0.001			<0.001
I-II	32	0		32	0	
III-IV	10	21		15	16	

Abbreviations: NPC naso-pharyngeal carcinoma; WHO World Healthy Organization; AJCC American Joint Committee on Cancer.

^a^*p*-value from Chi-square or Fisher exact test. Statistically significant (*p* < 0.05).

A DSS analysis using the Kaplan-Meier methods revealed that the prognosis of patients with high tumor expressions of FLJ10540 and osteopontin were significantly poorer than that of patients with low tumor expressions of FLJ10540 and osteopontin (*p<*0*.*001) (Figure 2B). Univariate analysis showed that DSS in this series was significantly associated with several clinico-pathological factors, including T (*p<*0*.*001), N (*p=*0*.*009), and TNM stage (*p=*0*.*001) (Table 3). Clinical prognosis, however, was not associated with age, gender or histological type. For multivariate analysis, however, T stage (*p<*0*.*001; hazard ratio = 5.091) remained significant independent prognostics factor of DSS for NPC patients. These results clearly indicate that the 5-year survival and clinical prognosis of NPC are associated with FLJ10540 and osteopontin expressions in NPC patients.

**Table 3:** **Disease-specific survival byCox regression analysesbetween the subgroups of various clincopathological parameters**

**Parameters**	**No**	**No of events**	**HR (95% CI^a^)**	***p-*value**^**b**^
Age (years)			1.695 (0.743-3.875)	0.211
<50	24	8		
≥ 50	39	19		
Gender			0.596 (0.225-1.575)	0.297
Male	47	22		
Female	16	5		
Histology			0.930 (0.431-.006)	0.854
WHO type II	37	16		
WHO type III	26	11		
Primary tumor (T)			5.091 (2.350-11.030)	<0.001
T1-T2	44	12		
T3-T4	19	15		
Nodal status (N)			2.780 (1.302-5.934)	0.009
N0-N1	41	13		
N2-N3	22	14		
AJCC staging			4.464 (1.879-10.607)	0.001
Stage I-II	31	7		
Stage III-IV	32	20		
FLJ10540			4.741 (2.177-10.326)	<0.001
Low expression	42	11		
High-expression	21	16		
Osteopontin			5.012 (2.315-10.850)	<0.001
Low expression	47	14		
High-expression	16	13		

Abbreviations: HR Hazard Ratio, WHO World Healthy Organization; AJCC American Joint Committee on Cancer.

^a^ 95% Wald Confidence Limits.

^b^ Associations determined by Cox regression analyses. Statistically significant (*p* < 0.05).

### Osteopontin triggered FLJ10540 expression in NPC cancer cells

**Figure 3:**
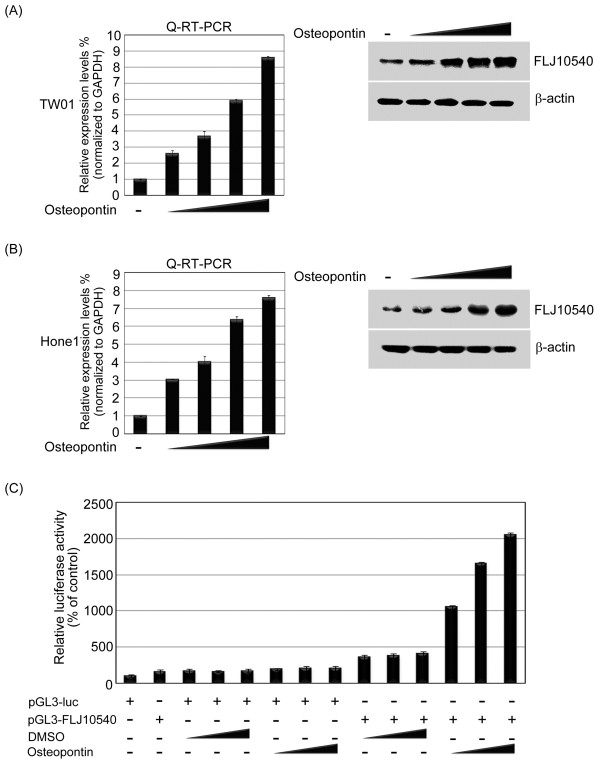
**FLJ10540 was up-regulated by osteopontin stimulation in human cancer cells.** (**A** and **B**, left panel) The mRNA expression level of *FLJ10540* was determined by Q-RT-PCR in TW01, and Hone1 cells with a dose-dependent manner of osteopontin. The results were normalized against the expression level of *GAPDH* mRNA in each osteopontin-treated cell. (**A** and **B**, right panel) The protein expression level of FLJ10540 was determined by Western blotting in TW01, and Hone1 cells. After treatment with various concentrations of osteopontin for 30 min, the total protein was extracted and the cell lysates (50 μg) of each treated cell were subjected to immunoblot analysis with anti-FLJ10540 and β-actin antibodies. β-actin was used as a control. Data are representative of three independent experiments done in triplicate. (**C**) Luciferase assays were done to detect promoter activity of FLJ10540 in transfected TW01 cells in the presence or absence of osteopontin. The luciferase activity in 1μg of cell lysate was normalized to β-galactosidase activity. Data are representative of three independent experiments done in triplicate.

As shown in the Figure 3A, the mRNA and protein expression levels of FLJ10540 were increased in an osteopontin dose-dependent manner in TW01 cells by Q-RT-PCR and Western blotting. Similar results were also obtained in the Hone1 cells (Figure 3B). Next, to investigate the role of osteopontin in regulating FLJ10540 transcription, we sought to determine whether FLJ10540 promoter activity could be regulated by osteopontin stimulation. We transfected the FLJ10540 promoter-luciferase constructs into TW01 cells following osteopontin stimulation in a dose-dependent manner. The data showed that a significant activation of the FLJ10540 promoter was detected under osteopontin stimulation (Figure 3C). These results illustrated that FLJ10540 is one of the downstream targets of the osteopontin signaling pathway in human NPC cancer cells.

### FLJ10540 transfectants alone or stimulated with osteopontin could promote cell growth in NPC

**Figure 4:**
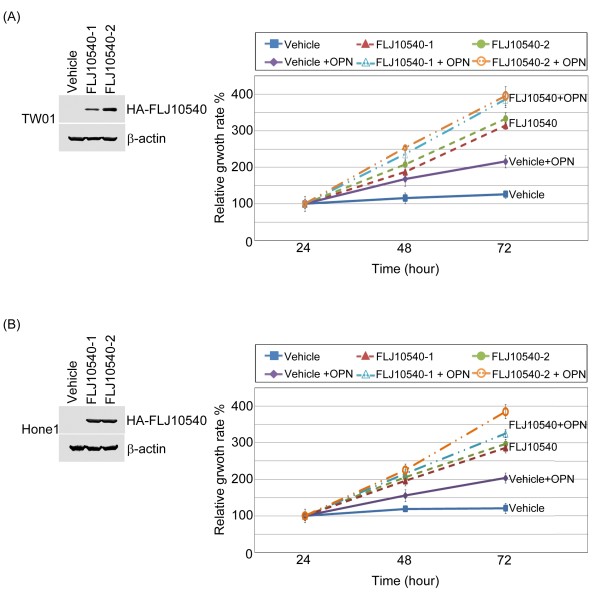
**Osteopontin enhanced FLJ10540-NPC transfectants to promote cell proliferation.** (**A** and **B**, left panel) HA-tagged FLJ10540-stable clones of TW01 and Hone1 cells were established. The cell lysates were subjected to an immunoblot analysis with an anti-HA antibody. (**A** and **B**, right panel) Vehicle-TW01/-Hone1 and FLJ10540-TW01/-Hone1 stable transfectants with or without osteopontin (OPN) stimulation were seeded into 96-well plates with 1.0% of FBS. The cells were cultured for 1–3 days followed by MTT assay (OD570) to quantitate cell growth. The data were normalized against the OD570 value on day 1 of each treatment. The growth curve of TW01 and Hone1 cells are shown as the mean ±s.d. of three independent experiments.

In Figure 2A, FLJ10540 expression is significantly higher in the NPC patients with a late tumor stage. This raises the possibility that FLJ10540 expression may promote tumor growth in NPC. Two different cell types were employed to address this question. First, TW01 and Hone1 cells stably expressing HA-tagged FLJ10540 were established (Figure 4A and B, left panel). FLJ10540-TW01 stable transfectants or vehicle control cells were seeded in 96-well plates to test whether they could grow in low serum medium. The results showed that FLJ10540-TW01 stable transfectants could promote cell growth in low-serum media compared to the vehicle control (Figure 4A, right panel). Similar results were also observed in FLJ10540-Hone1cells (Figure 4B, right panel). To determine whether osteopontin contributes to FLJ10540-mediated cell growth, FLJ10540-NPC transfectants, and vehicle control in the presence of osteopontin stimulation were seeded in 96-well plates to test cell proliferation. The results showed that FLJ10540-NPC stable transfectants in the presence of osteopontin grew faster than the vehicle control with osteopontin stimulation (Figure 4A and B, right panel). Furthermore, we also found that stimulated FLJ10540 transfectants with osteopontin was also growth faster than FLJ10540 stable cells alone. Taken together, these results suggest that FLJ10540 transfectants with or without osteopontin stimulation may decrease the requirement for serum, a characteristic of transformed cells.

### Knockdown of endogenous FLJ10540 by siRNA suppressed NPC cell growth

**Figure 5:**
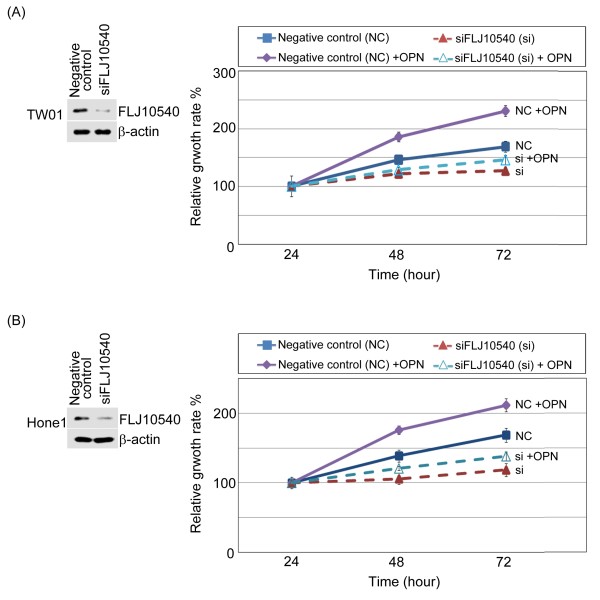
**Osteopontin reversed the cell growth inhibition of siFLJ10540-NPC.** (**A** and **B**, left panel) A negative control (NC) siRNA plus one different *FLJ10540* siRNA (siFLJ10540) were transfected into TW01 and Hone1 cells for 24 hours. After transfection, Western blotting was performed using anti-FLJ10540 and β-actin antibodies. (**A** and **B**, right panels) Using the same panel, the siFLJ10540 transfectants and NC with or without osteopontin (OPN) stimulation were seeded into 96-well plates with 1.0% of FBS. The cells were cultured for 1–3 days followed by MTT assay (OD570) to quantitate cell growth. The data were normalized against the OD570 value on day 1 of each treatment. The growth curve of TW01 and Hone1 cells are shown as the mean ±s.d. of three independent experiments.

We used a siRNA approach to inhibit endogenous expression of FLJ10540, and assayed for the growth rate of NPC cells. First, we transfected a chemically synthesized FLJ10540 siRNAs (siFLJ10540) and a negative control into TW01 and Hone1 for 24 hours, followed by Western blot analysis using antibodies against FLJ10540. The results showed a dramatic reduction in the FLJ10540 protein level with FLJ10540 siRNA (Figure 5A and B, left panel). Both TW01 and Hone1 siFLJ10540-transfected and negative control were determined the growth rates by MTT assay. The results demonstrated that the proliferation rate of FLJ10540 siRNA transfected cells was much slower than the cells transfected with negative siRNA (Figure 5A and B, right panel). Using the same panel, we also asked whether osteopontin encourages cell growth in depleted-FLJ10540 NPC cells. The result showed that FLJ10540-depleted NPC cells had a slight increased growth curve under osteopontin stimulation, compared to siFLJ10540 alone (Figure 5A and B, right panel). Take together these results strongly supports the idea that FLJ10540 plays a role in the cell proliferation of NPC cells.

### FLJ10540 and osteopontin synergistically induced the migration and invasion of NPC cancer cells

**Figure 6:**
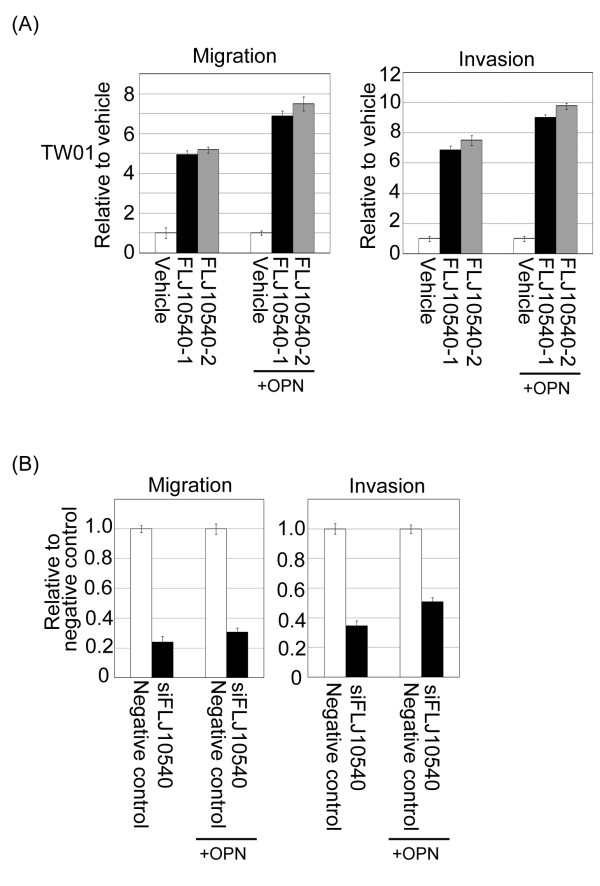
**FLJ10540 alone or increased FLJ10540, mediated by osteopontin stimulation modulated cell migration and invasion in NPC cells.** (**A** and **B**) For the migration assays, 5 × 10^3^ cells (Vehicle-TW01, FLJ10540-1-TW01, FLJ10540-2-TW01, negative control, and siFLJ10540-TW01) with or without osteopontin (OPN) stimulation were seeded into the top of a Transwell insert. After 24 hours, the cells on top were scraped, and the cells that had migrated to the bottom were fixed and stained with Giemsa. For the invasion assays, 1 × 10^4^ cells were seeded after the addition of Matrigel. The relative-fold migration/invasion values for the stable clones were normalized against the vehicle/negative control cells and are represented diagrammatically. All of the data represent the mean ± s.d. of three independent experiments.

FLJ10540 expression was higher in the NPC patients with lymph node metastasis (N+) than those that without lymph node metastasis (N-) (Figure 2A). We hypothesized that FLJ10540 may participate in the progression of metastasis in cells. FLJ10540-TW01 stable cells and vehicle control were seeded on a Transwell chamber to perform a motility assay for migration and invasion. Quantitatively speaking, the migration ability of FLJ10540-TW01 stable transfectants was approximately 4.8-5.5-fold higher than that of the vehicle control (Figure 6A, left panel). We next performed an invasion assay using a Matrigel-coated barrier filter. After 24 hours of incubation in the Matrigel-coated Transwell chamber, the TW01-FLJ10540 transfectants had invaded the basement membrane material and passed through the pores to reach the underside of the filter. Similarly, the TW01-FLJ10540 expressing cells showed a 6.8-7.8-fold higher invasive ability than the vehicle control cells (Figure 6A, right panel). The overexpressed FLJ10540 stable transfectants in the presence of osteopontin stimulation were analyzed the cell motility in a Transwell chamber. Again, FLJ10540 transfectants with osteopontin stimulation had a higher migration and invasion abilities than that the FLJ10540 alone or vehicle control (Figure 6A). Conversely, the motility of the NPC cells was strongly inhibited, while endogenous FLJ10540 was depleted by siRNA (Figure 6B). However, compared with siFLJ10540-NPC cells, the migratory and invasive abilities of siFLJ10540-NPC cells were slightly increased after cell-treated with osteopontin (Figure 6B). Taken together, these results strongly suggest that FLJ10540 is required for proper osteopontin-dependent signaling, and that it contributes to cell migration and invasion in NPC.

### Osteopontin-mediated FLJ10540 expression promoted cell proliferation and motility via CD44

**Figure 7:**
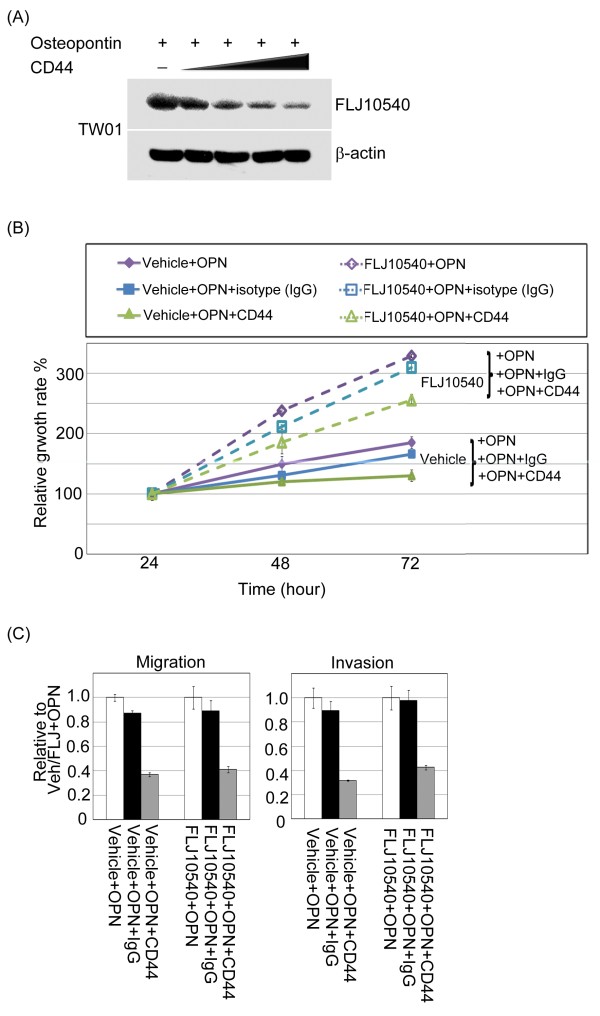
**CD44 not only participated in the FLJ10540 expression, but also modulated osteopontin/FLJ10540-elicited cell growth and motility in NPC cells.** (**A**) Serum-starved TW01 cells were pre-treated with or without various concentrations of anti-CD44 antibodies for 2 hours; the cells were then stimulated with 30 ng/ml osteopontin for 30 min. The total protein was extracted from TW01 cells and probed with antibodies against FLJ10540. β-actin was used as an internal loading control. (**B**) For the cell proliferation assay, vehicle-TW01, and FLJ10540-TW01 stable transfectants treated with or without osteopontin stimulation combined with anti-CD44 or IgG antibodies were seeded into 96-well plates with 1.0% FBS. The cells were cultured for 1–3 days followed by MTT assay (OD570) to quantitate cell growth. The data were normalized against the OD570 value on day 1 of each treatment. The growth curve of TW01 cells are shown as the mean ±s.d. of three independent experiments. (**C**) For the migration and invasion assay, vehicle-TW01 and FLJ10540-TW01 stable clones were pre-treated with or without anti-CD44 or IgG antibodies for 2 hours and seeded into the top of a Transwell insert with or without Matrigel and allowed to adhere for 12 hours. They were then incubated with or without osteopontin (30 ng/ml) for 3 hours. At the end of the assay, the cells on the topside were scraped, and the cells that migrated to the bottom were fixed and stained with Giemsa. All of the data represent the mean ±s.d. of three independent experiments.

We next asked whether the involvement of CD44 on the biological activities of osteopontin could be attributed to the modulation of FLJ10540 expression induced by osteopontin in NPC cancer cells. The CD44 receptors were blocked using neutralizing antibodies, and analyzed FLJ10540 protein expression in TW01 cells upon osteopontin stimulation. The results demonstrated that upregulation of FLJ10540 in the presence of osteopontin was inhibited by the simultaneous addition of CD44 antibodies in a dose-dependent manner (Figure 7A). Similar results were also observed in Hone1 cells (data not shown). To confirm the involvement of CD44 in mediating FLJ10540 function upon osteopontin stimulation, we treated FLJ10540-TW01 and vehicle cells with anti-CD44 blocking antibody or isotypes IgG antibody in the presence of osteopontin and monitored cell growth and motility. Anti-CD44 antibodies resulted in a significant inhibition of cell growth and motility by FLJ10540 stable cells in the presence of osteopontin stimulation, compared to FLJ10540 stable cells treated with isotype IgG antibody (Figure 7B, and C). Taken together, these results indicate that FLJ10540 is required for proper osteopontin/CD44-dependent signaling and that it contributes to cell proliferation and metastasis.

## Discussion

Our data illustrates that the up-regulation of FLJ10540 and osteopontin expressions are associated with a poor prognosis in NPC patients. It is worth noting that prognosis was not dependent on histologic type, but rather dependent on the expressions of FLJ10540 and osteopontin. FLJ10540 and osteopontin positively correlated with a poor prognosis in NPC patients, especially with T and N stages respectively. Because it is difficult to predict the prognosis of such patients, investigating FLJ10540 and osteopontin stainings together in NPC cancer cells may be helpful in determining the therapeutic strategy. Functionally, Ectopic expression of FLJ10540 enhanced cell growth, migration and invasion. Conversely, siRNA-mediated repression of FLJ10540 suppressed cell growth and motility in NPC. Moreover, blocked CD44 receptors by using CD44-specific antibodies, not only decreased the protein expression of FLJ10540 under osteopontin stimulation, but also suppressed FLJ10540-elicited NPC cell growth and metastasis, supporting the participation of FLJ10540 in the osteopontin/CD44 pathway. These findings suggest that FLJ10540 is not only a good prognostic indicator of NPC, but also a candidate for a molecular targeted therapy.

FLJ10540 overexpression is disclosed in a number of human cancers and cell lines, and has been correlated with tumor progression as well as decreased survival in patients with HCC, lung and oral cancer[21-23]. This suggests that FLJ10540 plays an important role in tumor formation. This is the first time we have shown that FLJ10540-NPC stable transfectants promoted cell growth at low serum levels. Conversely, suppressed endogenous FLJ10540 by FLJ10540-mediated siRNA inhibited cell growth, suggesting that FLJ10540 displays a characteristic associated with oncogenes in NPC. Furthermore, stimulation of FLJ10540-NPC stable cells by adding osteopontin could encourage cell growth faster than FLJ10540 alone or the vehicle control. Taken together, we suggest that FLJ10540, mediated by osteopontin stimulation, may play a significant role in tumor progression in NPC.

The metastatic spread of cancer cells is composed of multiple sequential steps such as dys-regulation of intercellular adhesion, degradation of the extracellular matrix, and increasing cell motility [31]. To the best of our knowledge, this is the first report to demonstrate that an increased FLJ10540 expression was not only positively correlated with clinical advanced N stage, but that is also promoted NPC cell migration and invasion *in vitro*. Moreover, the migratory and invasive abilities were abolished with knockdown endogenous FLJ10540 by siRNA in NPC cells. Importantly, FLJ10540-NPC transfectants stimulated by osteopontin enhanced cancer cell migration and invasion. Our results suggest that one mechanism by which FLJ10540 may promote tumor cell migration and invasion is through osteopontin regulation in NPC.

We re-arranged a gene expression microarray dataset and employed the concept of syn-expression to identify that *FLJ10540* is one of the downstream targets of *osteopontin* in NPC. Recently, it has been reported that osteopontin serves as a serum marker for some human tumor types [32]. Furthermore, an increased expression of osteopontin has been shown to correlate with disease outcome and patient survival. Osteopontin is able to engage receptors, including CD44, and thus may stimulate diverse signaling pathways and influence cellular events that in turn, favor tumorigenesis [33]. Using osteopontin-null mutant mice as a model system, osteopontin was demonstrated to have a role in the growth or survival of cancer cells [34]. However, the specific mechanisms by which osteopontin functions to promote malignant cell behavior have not been completely elucidated. In the present study, it is crucial to demonstrate that the mRNA and protein expression levels of FLJ10540 are positively correlated with osteopontin in NPC specimens. In addition, the expression level of FLJ10540 was upregulated in an osteopontin dose-dependent manner in NPC cell lines. The mechanisms responsible for the overexpression of FLJ10540 in malignant cancer cells are still not clear. Here, our results demonstrated that one of the important upstream regulators of FLJ10540 may be regulated by a cytokine, osteopontin, in NPC cancer cells. Furthermore, blockade of CD44 receptor caused significant inhibitions of FLJ10540-induced cell growth, migration and invasion in the presence of osteopontin stimulation in NPC cells. In this regard, our results show that CD44 provides an important link for understanding the role of osteoponint/FLJ10540 in cell proliferation and metastasis.

## Conclusions

FLJ10540 was regulated by osteopontin and plays an important role in NPC because increased FLJ10540 protein levels are observed to associate with advanced stage of NPC. In addition, FLJ10540 seems to contribute to tumor aggressiveness because high FLJ10540 protein levels are related to reduce patient survival. If the independence from established prognostic factors is demonstrated in larger clinical study, FLJ10540 genetic alterations in NPC may eventually be able to guide therapeutic strategies.

## Competing interests

The authors declare that they have no competing interests.

## Authors' contributions

CHC, LYS and CFH: collected the clinical data of patients, conceived the study design, carried out and coordinated immunohistochemical examinations of tumor specimens, and drafted the manuscript. LJS: NPC microarray analysis and interpretation. CYH: conceived the study design. SCH and CCH: participated in the interpretation of data and conducted immunohistochemistry analysis. WSW, YFY, HTT and WCC: performed the IHC staining and biochemical experiments. FMF: performed statistical data analysis. All authors have read and approved the final version of the manuscript.

## Supplementary Material

Additional file 1 The up-regulated and down-regualted genes involved in the development of human NPC. Click here for file
