# Supplementary material for: FLJ10540 is associated with tumor progression in nasopharyngeal carcinomas and contributes to nasopharyngeal cell proliferation, and metastasis via osteopontin/CD44 pathway
Source: J Transl Med. 2012 May 16;10:93. doi: 10.1186/1479-5876-10-93 (PMC3419101; doi:10.1186/1479-5876-10-93)
Supplement: Additional file 1 — The up-regulated and down-regualted genes involved in the development of human NPC. [file 1479-5876-10-93-S1.pdf]

Up-regulated genes

Gene Symbol	Probeset ID	Description
ADA	204639_at	adenosine deaminase
ADAM22	213411_at	ADAM metallopeptidase domain 22
ADAM23	244463_at	ADAM metallopeptidase domain 23
	213808_at	
ANGPT2	205572_at	angiopoietin 2
ANKRD29	238332_at	ankyrin repeat domain 29
ANLN	1552619_a_at	anillin, actin binding protein (scraps homolog, Drosophila)
	222608_s_at	
ASPM	219918_s_at	asp (abnormal spindle)-like, microcephaly associated (Drosophila)
ATAD2	228401_at	ATPase family, AAA domain containing 2
	222740_at	
ATP11C	226785_at	ATPase, Class VI, type 11C
BCL2A1	205681_at	BCL2-related protein A1
BIRC5	202095_s_at	baculoviral IAP repeat-containing 5 (survivin)
BRIP1	235609_at	BRCA1 interacting protein C-terminal helicase 1
BUB1B	203755_at	BUB1 budding uninhibited by benzimidazoles 1 homolog beta (yeast)
C10orf3	218542_at	chromosome 10 open reading frame 3 (FLJ10540)
C20orf42	218796_at	chromosome 20 open reading frame 42
	60474_at	
C5orf13	201310_s_at	chromosome 5 open reading frame 13
C6orf141	1552575_a_at	chromosome 6 open reading frame 141
	1554314_at	
CALD1	201617_x_at	caldesmon 1
	212077_at	
CCL2	216598_s_at	chemokine (C-C motif) ligand 2
CCL8	214038_at	chemokine (C-C motif) ligand 8
CCNB1	228729_at	cyclin B1
CCNE2	205034_at	cyclin E2
CD163	203645_s_at	CD163 antigen
CD200	209583_s_at	CD200 antigen
CDC2	203213_at	Cell division cycle 2, G1 to S and G2 to M
CDCA1	223381_at	cell division cycle associated 1
CENPF	209172_s_at	centromere protein F, 350/400ka (mitosin) ; centromere protein F, 350/400ka (mitosin)
CHI3L1	209395_at	chitinase 3-like 1 (cartilage glycoprotein-39)
CKS2	204170_s_at	CDC28 protein kinase regulatory subunit 2
CNTNAP2	219301_s_at	contactin associated protein-like 2
COL15A1	203477_at	collagen, type XV, alpha 1
COL1A1	1556499_s_at	collagen, type I, alpha 1
COL1A2	202404_s_at	collagen, type I, alpha 2
COL22A1	228873_at	collagen, type XXII, alpha 1
COL27A1	225288_at	Collagen, type XXVII, alpha 1
COL3A1	201852_x_at	collagen, type III, alpha 1 (Ehlers-Danlos syndrome type IV, autosomal dominant)
	215076_s_at	
	211161_s_at	
COL4A1	211980_at	collagen, type IV, alpha 1
COL4A2	211964_at	collagen, type IV, alpha 2
COL5A2	221730_at	collagen, type V, alpha 2
	221729_at	
COL8A1	226237_at	Collagen, type VIII, alpha 1
CSAG2	220445_s_at	CSAG family, member 2
CSE1L	201111_at	CSE1 chromosome segregation 1-like (yeast)
CSPG2	221731_x_at	chondroitin sulfate proteoglycan 2 (versican)
	204619_s_at	
CXCL10	204533_at	chemokine (C-X-C motif) ligand 10
CXCL11	210163_at	chemokine (C-X-C motif) ligand 11
	211122_s_at	
CXCL9	203915_at	chemokine (C-X-C motif) ligand 9
DEPDC1B	226980_at	DEP domain containing 1B
DMD	203881_s_at	dystrophin (muscular dystrophy, Duchenne and Becker types)
DNA2L	213647_at	DNA2 DNA replication helicase 2-like (yeast)
DTL	222680_s_at	denticleless homolog (Drosophila)
	218585_s_at	
ECT2	219787_s_at	epithelial cell transforming sequence 2 oncogene
ESCO2	235178_x_at	establishment of cohesion 1 homolog 2 (S. cerevisiae)
FANCL	227854_at	Fanconi anemia, complementation group L
FBN1	202766_s_at	fibrillin 1 (Marfan syndrome)
FIGNL1	222843_at	fidgetin-like 1

FLJ11029	228273_at	Hypothetical protein FLJ11029
FLJ12505	235343_at	Hypothetical protein FLJ12505
FLJ20641	227928_at	hypothetical protein FLJ20641
FMNL2	226184_at	formin-like 2
FN1	216442_x_at	fibronectin 1
	210495_x_at	
	212464_s_at	
	211719_x_at	
FRAS1	226145_s_at	Fraser syndrome 1
FZD7	203706_s_at	frizzled homolog 7 (Drosophila)
GAD1	205278_at	glutamate decarboxylase 1 (brain, 67kDa)
GALNT11	219013_at	UDP-N-acetyl-alpha-D-galactosamine:polypeptide N-acetylgalactosaminyltransferase 11 (GalNAc-T11)
GHR	205498_at	growth hormone receptor
GJA1	201667_at	gap junction protein, alpha 1, 43kDa (connexin 43)
GLS	221510_s_at	glutaminase
GMNN	218350_s_at	geminin, DNA replication inhibitor
GRB10	209409_at	growth factor receptor-bound protein 10
GREM1	218469_at	gremlin 1, cysteine knot superfamily, homolog (Xenopus laevis)
	218468_s_at	
GZMB	210164_at	granzyme B (granzyme 2, cytotoxic T-lymphocyte-associated serine esterase 1) ; granzyme B (granzyme 2,
HCAP-G	218663_at	chromosome condensation protein G
	218662_s_at	
HELLS	220085_at	helicase, lymphoid-specific
	242890_at	
HMMR	207165_at	hyaluronan-mediated motility receptor (RHAMM)
HOXA3	235521_at	homeo box A3
HOXC6	206858_s_at	homeo box C6
IFI44L	204439_at	interferon-induced protein 44-like
IFIT1	203153_at	interferon-induced protein with tetratricopeptide repeats 1 ; interferon-induced protein with tetratricopeptide
IFIT2	226757_at	interferon-induced protein with tetratricopeptide repeats 2
IFIT3	229450_at	interferon-induced protein with tetratricopeptide repeats 3
IMP-3	203820_s_at	IGF-II mRNA-binding protein 3
IMP-3	203819_s_at	IGF-II mRNA-binding protein 3
INHBA	227140_at	Inhibin, beta A (activin A, activin AB alpha polypeptide)
INSM1	206502_s_at	insulinoma-associated 1
IQGAP3	229490_s_at	IQ motif containing GTPase activating protein 3
ITGAV	236251_at	Integrin, alpha V (vitronectin receptor, alpha polypeptide, antigen CD51)
	232797_at	
ITGB6	226535_at	integrin, beta 6
KIAA0101	202503_s_at	KIAA0101
KIAA0286	212621_at	KIAA0286 protein
KIF11	204444_at	kinesin family member 11
KIF14	206364_at	kinesin family member 14
	236641_at	
KIF23	204709_s_at	kinesin family member 23
KLHL23	213610_s_at	kelch-like 23 (Drosophila)
KNTC2	204162_at	kinetochore associated 2
LAMB1	211651_s_at	laminin, beta 1 ; laminin, beta 1
	201505_at	
LGALS1	201105_at	lectin, galactoside-binding, soluble, 1 (galectin 1)
LHX2	206140_at	LIM homeobox 2
MAC30	212281_s_at	hypothetical protein MAC30
MAD2L1	203362_s_at	MAD2 mitotic arrest deficient-like 1 (yeast)
MASTL	228468_at	microtubule associated serine/threonine kinase-like
MCM8	224320_s_at	MCM8 minichromosome maintenance deficient 8 (S. cerevisiae)
MELK	204825_at	maternal embryonic leucine zipper kinase
MLF1IP	218883_s_at	MLF1 interacting protein
	229305_at	
MMP1	204475_at	matrix metallopeptidase 1 (interstitial collagenase)
MMP12	204580_at	matrix metallopeptidase 12 (macrophage elastase)
MMP3	205828_at	matrix metallopeptidase 3 (stromelysin 1, progelatinase)
MS4A4A	219607_s_at	membrane-spanning 4-domains, subfamily A, member 4
NEDD1	1560116_a_at	neural precursor cell expressed, developmentally down-regulated 1
	1552417_a_at	
	234984_at	
NFE2L3	204702_s_at	nuclear factor (erythroid-derived 2)-like 3
NID1	202007_at	nidogen 1
NOV	214321_at	nephroblastoma overexpressed gene
NPL	221210_s_at	N-acetylneuraminate pyruvate lyase (dihydrodipicolinate synthase) ; N-acetylneuraminate pyruvate lyase (di

	240440_at	
NRXN1	228547_at	neurexin 1
NTRK2	221796_at	neurotrophic tyrosine kinase, receptor, type 2
NUSAP1	218039_at	nucleolar and spindle associated protein 1
OVOS2	228245_s_at	ovostatin 2
PAICS	201014_s_at	phosphoribosylaminoimidazole carboxylase, phosphoribosylaminoimidazole succinocarboxamide synthetase
PAPSS2	203058_s_at	3'-phosphoadenosine 5'-phosphosulfate synthase 2
	203060_s_at	
PBK	219148_at	PDZ binding kinase
PCOLCE2	219295_s_at	procollagen C-endopeptidase enhancer 2
PLA2G7	206214_at	phospholipase A2, group VII (platelet-activating factor acetylhydrolase, plasma) ; phospholipase A2, group
PLAU	205479_s_at	plasminogen activator, urokinase
PMAIP1	204285_s_at	phorbol-12-myristate-13-acetate-induced protein 1
	204286_s_at	
POSTN	1555778_a_at	periostin, osteoblast specific factor
	210809_s_at	
PRC1	218009_s_at	protein regulator of cytokinesis 1
PRRX1	238852_at	Paired related homeobox 1
PSAT1	223062_s_at	phosphoserine aminotransferase 1
PSF1	206102_at	DNA replication complex GINS protein PSF1
PTGS2	1554997_a_at	prostaglandin-endoperoxide synthase 2 (prostaglandin G/H synthase and cyclooxygenase)
	204748_at	
RAD51AP1	204146_at	RAD51 associated protein 1
RCN1	201063_at	reticulocalbin 1, EF-hand calcium binding domain
ROBO1	1558714_at	Roundabout, axon guidance receptor, homolog 1 (Drosophila)
RRM2	201890_at	ribonucleotide reductase M2 polypeptide
RSAD2	242625_at	radical S-adenosyl methionine domain containing 2
SCNN1G	241436_at	sodium channel, nonvoltage-gated 1, gamma
SLC39A14	212110_at	solute carrier family 39 (zinc transporter), member 14
SOCS1	210001_s_at	suppressor of cytokine signaling 1
SPARC	200665_s_at	secreted protein, acidic, cysteine-rich (osteonectin) ; secreted protein, acidic, cysteine-rich (osteonectin)
SPP1	209875_s_at	secreted phosphoprotein 1 (osteopontin, bone sialoprotein I, early T-lymphocyte activation 1)
STAR	204548_at	steroidogenic acute regulator
STK6	208079_s_at	serine/threonine kinase 6
SULF1	212353_at	sulfatase 1
SYNPO2	227662_at	synaptopodin 2
	225895_at	
TMPO	203432_at	thymopoietin
TNFAIP6	206025_s_at	tumor necrosis factor, alpha-induced protein 6
	206026_s_at	
TNFSF4	207426_s_at	tumor necrosis factor (ligand) superfamily, member 4 (tax-transcriptionally activated glycoprotein 1, 34kDa
TOP2A	201291_s_at	topoisomerase (DNA) II alpha 170kDa
	201292_at	
TYMS	202589_at	thymidylate synthetase
VRK2	243299_at	Vaccinia related kinase 2
XG	1554062_at	Xg blood group (pseudoautosomal boundary-divided on the X chromosome)
ZIC2	223642_at	Zic family member 2 (odd-paired homolog, Drosophila)
ZNF367	229551_x_at	zinc finger protein 367
	1563414_at	Full length insert cDNA clone YW28F05
	229242_at	Transcribed locus
	230782_at	CDNA FLJ33419 fis, clone BRACE2019877
	227498_at	CDNA FLJ11723 fis, clone HEMBA1005314
	227350_at	CDNA FLJ11381 fis, clone HEMBA1000501
	242881_x_at	LOC440156
	230312_at	CDNA FLJ45482 fis, clone BRTHA2001953
	227623_at	MRNA; cDNA DKFZp313C0240 (from clone DKFZp313C0240)
	227921_at	

Down-regulated genes

Gene Symbol	Probeset ID	Description
ABCA13	1553605_a_at	ATP-binding cassette, sub-family A (ABC1), member 13
	1553604_at	
ADH1A ; ADH1B ; A	206262_at	alcohol dehydrogenase 1A (class I), alpha polypeptide ; alcohol dehydrogenase IB (class I), beta polypeptid
ADH7	210505_at	alcohol dehydrogenase 7 (class IV), mu or sigma polypeptide
ADSSL1	226325_at	adenylosuccinate synthase like 1
AGBL2	220390_at	ATP/GTP binding protein-like 2
AGR2	209173_at	anterior gradient 2 homolog (Xenopus laevis)

AK7	1553734_at	adenylate kinase 7
AKAP14	237281_at	A kinase (PRKA) anchor protein 14
	237282_s_at	
AKR1C3	209160_at	aldo-keto reductase family 1, member C3 (3-alpha hydroxysteroid dehydrogenase, type II)
ALDH1A1	212224_at	aldehyde dehydrogenase 1 family, member A1
ALDH1A3	203180_at	aldehyde dehydrogenase 1 family, member A3
ALDH3A1	205623_at	aldehyde dehydrogenase 3 family, memberA1
ALDH3B1	205640_at	aldehyde dehydrogenase 3 family, member B1
AMY1A ; AMY1B ;	208498_s_at	amylase, alpha 1A; salivary ; amylase, alpha 1B; salivary ; amylase, alpha 1C; salivary ; amylase, alpha 2A;
ANXA1	201012_at	annexin A1
AQP3	39248_at	aquaporin 3
ARHGAP18	225166_at	Rho GTPase activating protein 18
ARMC2	223866_at	armadillo repeat containing 2
ARMC3	240275_at	armadillo repeat containing 3
ARMC4	223794_at	armadillo repeat containing 4
ATP12A	207367_at	ATPase, H+/K+ transporting, nongastric, alpha polypeptide
B3GNT7	1555963_x_at	UDP-GlcNAc:betaGal beta-1,3-N-acetylglucosaminyltransferase 7
	1555962_at	
BANK1	219667_s_at	B-cell scaffold protein with ankyrin repeats 1
BCAS1	204378_at	breast carcinoma amplified sequence 1
BCMP11	228241_at	breast cancer membrane protein 11
C10orf63	237314_at	chromosome 10 open reading frame 63
C10orf79	231084_at	chromosome 10 open reading frame 79
C10orf81	219857_at	chromosome 10 open reading frame 81
C14orf45	220173_at	chromosome 14 open reading frame 45
C14orf78	212992_at	chromosome 14 open reading frame 78
C19orf33	223631_s_at	chromosome 19 open reading frame 33
C1orf102	227359_at	chromosome 1 open reading frame 102
C1orf110	1554960_at	chromosome 1 open reading frame 110
C1orf114	206721_at	chromosome 1 open reading frame 114
C1orf116	219476_at	chromosome 1 open reading frame 116
	228865_at	
C1orf173	229973_at	chromosome 1 open reading frame 173
C1orf192	231077_at	chromosome 1 open reading frame 192
C1orf87	236710_at	chromosome 1 open reading frame 87
C1orf88	228100_at	chromosome 1 open reading frame 88
C20orf114	226067_at	chromosome 20 open reading frame 114
C20orf85	229542_at	chromosome 20 open reading frame 85
C3orf15	236222_at	chromosome 3 open reading frame 15
C6orf103	220614_s_at	chromosome 6 open reading frame 103
C6orf118	232777_s_at	chromosome 6 open reading frame 118
C6orf165	230273_at	chromosome 6 open reading frame 165
C6orf206	230695_s_at	chromosome 6 open reading frame 206
C6orf32	209829_at	chromosome 6 open reading frame 32
C8orf47	1552390_a_at	chromosome 8 open reading frame 47
C9orf116	59437_at	chromosome 9 open reading frame 116
	221946_at	
C9orf18	229976_at	chromosome 9 open reading frame 18
C9orf24	229012_at	chromosome 9 open reading frame 24
C9orf26	209821_at	chromosome 9 open reading frame 26 (NF-HEV)
CALML4	221879_at	calmodulin-like 4
	64408_s_at	
CAPN14	1557321_a_at	calpain 14
CAPS	231729_s_at	calcyphosine
	226424_at	
	231728_at	
CAPS2	224370_s_at	calcyphosine 2 ; calcyphosine 2
CAPSL	236085_at	calcyphosine-like
CASC1	220168_at	cancer susceptibility candidate 1
CCDC11	1552326_a_at	coiled-coil domain containing 11
	1552325_at	
CCDC17	236320_at	coiled-coil domain containing 17
CCDC19	220308_at	coiled-coil domain containing 19
CDH26	232306_at	cadherin-like 26
	233663_s_at	
CDS1	205709_s_at	CDP-diacylglycerol synthase (phosphatidate cytidylyltransferase) 1
CEACAM5	201884_at	carcinoembryonic antigen-related cell adhesion molecule 5
CEACAM6	211657_at	carcinoembryonic antigen-related cell adhesion molecule 6 (non-specific cross reacting antigen) ; carcinoem
	203757_s_at	

CES1	209616_s_at	carboxylesterase 1 (monocyte/macrophage serine esterase 1)
CGI-38	218876_at	brain specific protein ; brain specific protein
CH25H	206932_at	cholesterol 25-hydroxylase
CHL1	204591_at	cell adhesion molecule with homology to L1CAM (close homolog of L1)
CHST5	219182_at	carbohydrate (N-acetylglucosamine 6-O) sulfotransferase 5
	64900_at	
CHST6	223786_at	carbohydrate (N-acetylglucosamine 6-O) sulfotransferase 6
CHST9	223737_x_at	carbohydrate (N-acetylgalactosamine 4-0) sulfotransferase 9
	224400_s_at	
CKB	200884_at	creatine kinase, brain
CLCA2	217528_at	chloride channel, calcium activated, family member 2
CLCA4	220026_at	chloride channel, calcium activated, family member 4
CLDN10	205328_at	claudin 10
CLDN23	228707_at	claudin 23
CLIC3	219529_at	chloride intracellular channel 3
CLIC6	227742_at	chloride intracellular channel 6
CLMN	221042_s_at	calmin (calponin-like, transmembrane)
CLU	208792_s_at	clusterin (complement lysis inhibitor, SP-40,40, sulfated glycoprotein 2, testosterone-repressed prostate mes
	208791_at	
	222043_at	
CNFN	224329_s_at	cornifelin ; cornifelin
CR2	205544_s_at	complement component (3d/Epstein Barr virus) receptor 2
CRIP1	205081_at	cysteine-rich protein 1 (intestinal)
CTGF	209101_at	connective tissue growth factor
CXCL1	204470_at	chemokine (C-X-C motif) ligand 1 (melanoma growth stimulating activity, alpha)
CXCL14	218002_s_at	chemokine (C-X-C motif) ligand 14
	222484_s_at	
CXorf41	231389_at	chromosome X open reading frame 41
CYP4B1	210096_at	cytochrome P450, family 4, subfamily B, polypeptide 1
DAF	201925_s_at	decay accelerating factor for complement (CD55, Cromer blood group system)
	201926_s_at	
	1555950_a_at	
DENND2C	230769_at	DENN/MADD domain containing 2C
DHCR24	200862_at	24-dehydrocholesterol reductase
DHRS9	219799_s_at	dehydrogenase/reductase (SDR family) member 9
	224009_x_at	
	223952_x_at	
DKFZP434H0115	223924_at	hypothetical protein DKFZp434H0115
DKFZp666G057	1556158_at	hypothetical protein DKFZp666G057
DKFZp761N1114	229254_at	hypothetical protein DKFZp761N1114
DNAH5	232381_s_at	dynein, axonemal, heavy polypeptide 5
DNAH7	214222_at	dynein, axonemal, heavy polypeptide 7
DNAH9	240857_at	dynein, axonemal, heavy polypeptide 9
	207959_s_at	
DNAI2	220636_at	dynein, axonemal, intermediate polypeptide 2
DNAJA4	225061_at	DnaJ (Hsp40) homolog, subfamily A, member 4
DNALI1	227081_at	dynein, axonemal, light intermediate polypeptide 1
	205186_at	
DNCH2	219469_at	dynein, cytoplasmic, heavy polypeptide 2
DNCL2B	238116_at	dynein, cytoplasmic, light polypeptide 2B
DNER	226281_at	delta-notch-like EGF repeat-containing transmembrane
DUOX1	219597_s_at	dual oxidase 1
DUOX2	219727_at	dual oxidase 2
DUSP1	201041_s_at	dual specificity phosphatase 1
DYX1C1	235273_at	dyslexia susceptibility 1 candidate 1
ECRG4	223623_at	esophageal cancer related gene 4 protein
EFHB	239477_at	EF-hand domain family, member B
EFHC1	219833_s_at	EF-hand domain (C-terminal) containing 1
EFHC2	220591_s_at	EF-hand domain (C-terminal) containing 2
ELF3	229842_at	E74-like factor 3 (ets domain transcription factor, epithelial-specific)
	210827_s_at	
	201510_at	
ELL3	219518_s_at	elongation factor RNA polymerase II-like 3
EPAS1	200878_at	endothelial PAS domain protein 1
EPPK1	232164_s_at	epiplakin 1
	232165_at	
F3	204363_at	coagulation factor III (thromboplastin, tissue factor)
FAM3B	227194_at	family with sequence similarity 3, member B
FAM3D	227676_at	family with sequence similarity 3, member D

FAM81B	240065_at	family with sequence similarity 81, member B
FANK1	232968_at	fibronectin type III and ankyrin repeat domains 1
FBXO15	231472_at	F-box protein 15
FCRL4	224403_at	Fc receptor-like 4 ; Fc receptor-like 4
FLJ13841	219995_s_at	hypothetical protein FLJ13841
FLJ20366	218692_at	hypothetical protein FLJ20366
FLJ22662	218454_at	hypothetical protein FLJ22662
FLJ23049	220269_at	hypothetical protein FLJ23049
FLJ23514	220389_at	hypothetical protein FLJ23514
FLJ23577	232745_x_at	KPL2 protein
FLJ23834	235650_at	hypothetical protein FLJ23834
FLJ32926	233157_x_at	hypothetical protein FLJ32926
FLJ34512	236745_at	hypothetical protein FLJ34512
FLJ35834	1568924_a_at	hypothetical protein FLJ35834
FLJ37927	240161_s_at	CDC20-like protein
FLJ40083	243758_at	hypothetical protein FLJ40083
FLJ40427	243802_at	hypothetical protein FLJ40427
FLJ40873	1553635_s_at	Hypothetical protein LOC200132
FLJ40919	1556711_at	hypothetical protein FLJ40919
FLJ46154	232603_at	FLJ46154 protein
FLJ46266	1568606_at	FLJ46266 protein
FLJ46675	239499_at	FLJ46675 protein
FLJ90575	238682_at	hypothetical protein FLJ90575
GABRP	205044_at	gamma-aminobutyric acid (GABA) A receptor, pi
GCNT3	219508_at	glucosaminyl (N-acetyl) transferase 3, mucin type
GDA	224209_s_at	guanine deaminase
GNE	205042_at	glucosamine (UDP-N-acetyl)-2-epimerase/N-acetylmannosamine kinase
GPR110	235988_at	G protein-coupled receptor 110
GSTA1	203924_at	glutathione S-transferase A1
GSTA3	222102_at	glutathione S-transferase A3
GULP1	204237_at	GULP, engulfment adaptor PTB domain containing 1
	204235_s_at	
H19	224646_x_at	H19, imprinted maternally expressed untranslated mRNA
H2AFJ	225245_x_at	H2A histone family, member J
HOP	211597_s_at	homeodomain-only protein ; homeodomain-only protein
HRASLS2	221122_at	HRAS-like suppressor 2
HS3ST6 ; LOC34246	239547_at	heparan sulfate (glucosamine) 3-O-sulfotransferase 6 ; similar to c439A6.1 (novel protein similar to heparan
HSPB8	221667_s_at	heat shock 22kDa protein 8
HSPC065	222890_at	HSPC065 protein
HYDIN	232984_at	hydrocephalus inducing
IGHD	213674_x_at	immunoglobulin heavy constant delta
IGHM	212827_at	immunoglobulin heavy constant mu ; immunoglobulin heavy constant mu
IPO9	225728_at	Importin 9
IQCA	238584_at	IQ motif containing with AAA domain
IQCD	1552540_s_at	IQ motif containing D
	221185_s_at	
KCNE1	236407_at	potassium voltage-gated channel, Isk-related family, member 1
KCNRG	240288_at	potassium channel regulator
KIAA0500	213839_at	KIAA0500 protein
KIF21A	226003_at	kinesin family member 21A
KRT13	207935_s_at	keratin 13
KRT14	209351_at	keratin 14 (epidermolysis bullosa simplex, Dowling-Meara, Koebner)
KRT23	218963_s_at	keratin 23 (histone deacetylase inducible)
KRT4	213240_s_at	keratin 4
KRT7	209016_s_at	keratin 7
LCN2	212531_at	lipocalin 2 (oncogene 24p3)
LMO2	204249_s_at	LIM domain only 2 (rhombotin-like 1)
LOC127003	231044_at	similar to CG5435-PA
LOC128153	230763_at	hypothetical protein BC014608
LOC129881	236909_at	hypothetical LOC129881
LOC132203	239150_at	similar to hypothetical protein A430083B19
LOC134121	239722_at	hypothetical protein LOC134121
LOC136288	1557636_a_at	hypothetical protein LOC136288
LOC138255	243610_at	OTTHUMP00000021439
LOC151438	1560679_at	hypothetical protein LOC151438
LOC222171	226961_at	hypothetical protein LOC222171
LOC222967 ; LOC28	1557417_s_at	hypothetical protein LOC222967 ; hypothetical protein LOC285927
LOC283663	230245_s_at	hypothetical protein LOC283663
LOC401551	243900_at	similar to hypothetical protein FLJ25955

LOC441054	236915_at	hypothetical protein LOC441054
LOC90557 ; DKFZp4	227966_s_at	hypothetical protein BC016861 ; hypothetical protein DKFZp434E2321
LRP11	225060_at	low density lipoprotein receptor-related protein 11
LRRC34	236918_s_at	leucine rich repeat containing 34
	236917_at	
LRRC46	230601_s_at	leucine rich repeat containing 46
LRRC48	208140_s_at	leucine rich repeat containing 48 ; leucine rich repeat containing 48
LTB4DH	231897_at	leukotriene B4 12-hydroxydehydrogenase
LTF	202018_s_at	lactotransferrin
MAL	204777_s_at	mal, T-cell differentiation protein
MDAC1	1552594_at	MDAC1
MDH1B	1558077_s_at	malate dehydrogenase 1B, NAD (soluble)
MGC13040	224463_s_at	hypothetical protein MGC13040 ; hypothetical protein MGC13040
	241198_s_at	
MGC14839	238805_at	similar to RIKEN cDNA 2310030G06 gene
MGC16186	239733_at	hypothetical protein MGC16186
MGC16372	231133_at	CG10958-like
MGC26733	1562371_s_at	hypothetical protein MGC26733
MGC26963	227038_at	hypothetical protein MGC26963
MGC35043	231565_at	hypothetical protein MGC35043
MGC39581	237020_at	hypothetical protein MGC39581
MGC42367	228067_at	similar to 2010300C02Rik protein
MGC72075	228600_x_at	hypothetical protein MGC72075
MGLL	225102_at	monoglyceride lipase
MLF1	204784_s_at	myeloid leukemia factor 1
MNS1	219703_at	meiosis-specific nuclear structural 1
MOPT	226790_at	protein containing single MORN motif in testis
MPP7	238778_at	membrane protein, palmitoylated 7 (MAGUK p55 subfamily member 7)
MS4A1	228592_at	membrane-spanning 4-domains, subfamily A, member 1
	228599_at	
MS4A8B	224355_s_at	membrane-spanning 4-domains, subfamily A, member 8B ; membrane-spanning 4-domains, subfamily A, m
MSLN	204885_s_at	mesothelin
MSMB	207430_s_at	microseminoprotein, beta-
	210297_s_at	
MUC1	213693_s_at	mucin 1, transmembrane
	207847_s_at	
MUC13	218687_s_at	mucin 13, epithelial transmembrane
MUC15	227238_at	mucin 15
MUC16	220196_at	mucin 16
MUC20	226622_at	mucin 20
	231941_s_at	
MUC4	217109_at	mucin 4, tracheobronchial
	217110_s_at	
	204895_x_at	
MUC5AC	214385_s_at	mucin 5, subtypes A and C, tracheobronchial/gastric
	214303_x_at	
NBEA	221207_s_at	neurobeachin
	226439_s_at	
NEBL	203961_at	nebulette
NME5	206197_at	non-metastatic cells 5, protein expressed in (nucleoside-diphosphate kinase)
NQO1	210519_s_at	NAD(P)H dehydrogenase, quinone 1
	201467_s_at	
	201468_s_at	
NUCB2	203675_at	nucleobindin 2
NYD-SP14	223962_at	NYD-SP14 protein
PACRG	214204_at	PARK2 co-regulated
PAX5	221969_at	Paired box gene 5 (B-cell lineage specific activator)
PCSK5	205559_s_at	proprotein convertase subtilisin/kexin type 5
PI3	203691_at	peptidase inhibitor 3, skin-derived (SKALP) ; peptidase inhibitor 3, skin-derived (SKALP)
	41469_at	
PIGR	226147_s_at	polymeric immunoglobulin receptor
	229659_s_at	
PLAC8	219014_at	placenta-specific 8
PLEKHH1	225726_s_at	pleckstrin homology domain containing, family H (with MyTH4 domain) member 1
PPL	203407_at	periplakin
PROM1	204304_s_at	prominin 1
PRSS23	226279_at	protease, serine, 23
PRSS23	202458_at	protease, serine, 23
PTPRZ1	204469_at	protein tyrosine phosphatase, receptor-type, Z polypeptide 1

RBM24	235004_at	RNA binding motif protein 24
RDHE2	238017_at	epidermal retinal dehydrogenase 2
RFX2	226872_at	regulatory factor X, 2 (influences HLA class II expression)
RHCG	219554_at	Rhesus blood group, C glycoprotein
RNASE4	213397_x_at	ribonuclease, RNase A family, 4
ROPN1L	223609_at	ropporin 1-like
RRAD	204803_s_at	Ras-related associated with diabetes
	204802_at	
RSHL3	233071_at	radial spokehead-like 3
S100A2	204268_at	S100 calcium binding protein A2
S100A8	214370_at	S100 calcium binding protein A8 (calgranulin A)
S100A9	203535_at	S100 calcium binding protein A9 (calgranulin B)
S100P	204351_at	S100 calcium binding protein P
SAA1	214456_x_at	serum amyloid A1
SAA1 ; SAA2	208607_s_at	serum amyloid A1 ; serum amyloid A1 ; serum amyloid A2 ; serum amyloid A2
SAA4	207096_at	serum amyloid A4, constitutive
SCEL	206884_s_at	sciellin
	232056_at	
SCGB1A1	205725_at	secretoglobin, family 1A, member 1 (uteroglobin)
SCGB2A1	205979_at	secretoglobin, family 2A, member 1
SCIN	1552365_at	scinderin
SCNN1A	203453_at	sodium channel, nonvoltage-gated 1 alpha
SELENBP1	214433_s_at	selenium binding protein 1 ; selenium binding protein 1
SERPINB1	228726_at	Serpin peptidase inhibitor, clade B (ovalbumin), member 1
SERPINB11	1552463_at	serpin peptidase inhibitor, clade B (ovalbumin), member 11
SERPINB13	211361_s_at	serpin peptidase inhibitor, clade B (ovalbumin), member 13
	217272_s_at	
SERPINB2	204614_at	serpin peptidase inhibitor, clade B (ovalbumin), member 2
SERPINB3	209720_s_at	serpin peptidase inhibitor, clade B (ovalbumin), member 3
	209719_x_at	
SERPINB4	210413_x_at	serpin peptidase inhibitor, clade B (ovalbumin), member 4
SERPINB7	206421_s_at	serpin peptidase inhibitor, clade B (ovalbumin), member 7
SLC15A2	205316_at	Solute carrier family 15 (H+/peptide transporter), member 2
SLC16A5	206600_s_at	solute carrier family 16 (monocarboxylic acid transporters), member 5
SLC16A9	227506_at	solute carrier family 16 (monocarboxylic acid transporters), member 9
SLC22A16	232232_s_at	solute carrier family 22 (organic cation transporter), member 16
SLC22A4	205896_at	solute carrier family 22 (organic cation transporter), member 4
SLC27A2	205768_s_at	solute carrier family 27 (fatty acid transporter), member 2
	205769_at	
SLC2A10	221024_s_at	solute carrier family 2 (facilitated glucose transporter), member 10 ; solute carrier family 2 (facilitated gluco
SLC44A4	205597_at	solute carrier family 44, member 4
SLC6A14	219795_at	solute carrier family 6 (amino acid transporter), member 14
SLPI	203021_at	secretory leukocyte peptidase inhibitor
SORBS2	204288_s_at	sorbin and SH3 domain containing 2
SPA17	205406_s_at	sperm autoantigenic protein 17
SPAG17	233516_s_at	sperm associated antigen 17
SPAG6	210033_s_at	sperm associated antigen 6
	210032_s_at	
SPATA18	229331_at	spermatogenesis associated 18 homolog (rat)
SPINK5	205185_at	serine peptidase inhibitor, Kazal type 5
SPRR1A	213796_at	small proline-rich protein 1A
SPRR3	218990_s_at	small proline-rich protein 3
SRGAP3	1559333_at	SLIT-ROBO Rho GTPase activating protein 3
ST6GALNAC1	227725_at	ST6 (alpha-N-acetyl-neuraminyl-2,3-beta-galactosyl-1,3)-N-acetylgalactosaminide alpha-2,6-sialyltransferas
STOML3	1553794_at	stomatin (EPB72)-like 3
SYTL2	232914_s_at	synaptotagmin-like 2
TACSTD2	202286_s_at	tumor-associated calcium signal transducer 2
TEKT1	239216_at	tektin 1
TEKT2	210323_at	tektin 2 (testicular)
TJP3	35148_at	tight junction protein 3 (zona occludens 3)
TMC5	240304_s_at	Transmembrane channel-like 5
	219580_s_at	
	222904_s_at	
TMEM45B	230323_s_at	transmembrane protein 45B
	226226_at	
TNRC9	214774_x_at	trinucleotide repeat containing 9
TP53INP2	224836_at	tumor protein p53 inducible nuclear protein 2
TPPP	230104_s_at	brain-specific protein p25 alpha
TSGA10	223838_at	testis specific, 10

TSGA2	230093_at	testis specific A2 homolog (mouse)
TSPAN1	209114_at	tetraspanin 1
TTC18	229170_s_at	tetratricopeptide repeat domain 18
	229169_at	
TTC9	213172_at	tetratricopeptide repeat domain 9
	213174_at	
TUBB2	204141_at	tubulin, beta 2
UBXD3	238657_at	UBX domain containing 3
UGT1A10 ; UGT1A8	215125_s_at	UDP glucuronosyltransferase 1 family, polypeptide A10 ; UDP glucuronosyltransferase 1 family, polypeptic
UNQ473	226960_at	DMC
UPK1B	210065_s_at	uroplakin 1B
	210064_s_at	
URB	225242_s_at	steroid sensitive gene 1
UTP14C	237168_at	UTP14, U3 small nucleolar ribonucleoprotein, homolog C (yeast)
VNN3	220528_at	vanin 3
WDR49	1554298_a_at	WD repeat domain 49
WDR66	230193_at	WD repeat domain 66
	1555007_s_at	
WDR69	242162_at	WD repeat domain 69
WDR78	229816_at	WD repeat domain 78
WDRPUH	239916_at	WD40-repear protein upregulated in HCC
WFDC2	203892_at	WAP four-disulfide core domain 2
YSK4	1555804_a_at	Yeast Sps1/Ste20-related kinase 4 (S. cerevisiae)
ZD52F10	226926_at	dermokine
ZMYND10	205714_s_at	zinc finger, MYND-type containing 10
ZMYND12	223636_at	zinc finger, MYND-type containing 12
	238720_at	
	236489_at	Transcribed locus
	239776_at	MRNA; cDNA DKFZp686G0585 (from clone DKFZp686G0585)
	1556003_a_at	
	1562921_at	
	241898_at	Transcribed locus, moderately similar to XP_517655.1 PREDICTED: similar to KIAA0825 protein [Pan tro
	241310_at	Transcribed locus
	243803_at	
	222271_at	Transcribed locus
	240303_at	CDNA FLJ44282 fis, clone TRACH2003516
	240293_at	
	236666_s_at	Similar to leucine rich repeat containing 10
	243780_at	CDNA FLJ46553 fis, clone THYMU3038879
	224348_s_at	
	213929_at	Homo sapiens, Similar to likely ortholog of yeast ARV1, clone IMAGE:4733238, mRNA
	229810_at	Transcribed locus, weakly similar to XP_496299.1 PREDICTED: hypothetical protein LOC148206 [Homo s
	231582_at	Transcribed locus
	235651_at	Transcribed locus, moderately similar to NP_872301.1 hypothetical protein FLJ25224 [Homo sapiens]
	244313_at	Transcribed locus
	230043_at	
	1568906_at	CDNA clone IMAGE:4820483
